# From movement to mood regulation: exercise-based digital interventions using mobile health and ecological momentary assessment for mental health and cognitive function in older adults: a narrative review

**DOI:** 10.3389/fpsyt.2026.1895581

**Published:** 2026-09-30

**Authors:** Wenjie Wu

**Affiliations:** Physical Institute of Suzhou University of Science and Technology, Suzhou, Jiangsu, China

**Keywords:** ecological momentary assessment, exercise-based digital interventions, mobile health, mood regulation, movement, older adults

## Abstract

This narrative review aims to synthesize current evidence on the integration of exercise with mobile health technologies and ecological momentary assessment or intervention (EMA/EMI) to promote mental and cognitive well-being in older adults. Given the rapid aging of the global population and the high attrition rates in traditional exercise programs, evaluating these digital, real-time modalities is of significant scientific interest. To capture the current evidence base, a comprehensive literature search was conducted across PubMed/MEDLINE, Scopus, Web of Science, and Google Scholar. Peer-reviewed articles encompassing randomized controlled trials, pilot studies, and observational designs that evaluated digitally supported exercise interventions for mental and cognitive health in adults aged 50 and older were selected for this narrative synthesis. Recent empirical investigations have demonstrated the increasing application of digital modalities, including wearable activity monitors, smartphone applications, and sensor-initiated evaluations, within exercise regimens to facilitate the real-time tracking and adjustment of physical activity levels. The potential benefits of these technological interventions on mental and cognitive outcomes may operate via dynamic behavioral and neurophysiological feedback processes. The evidence generally suggests that elevated levels of physical activity correlate positively with enhanced mood, reductions in depressive symptoms, and improved cognitive functioning, whereas sedentary behaviors and fatigue are associated with detrimental affective outcomes. Significantly, studies employing EMA elucidate considerable intra-individual variability, indicating that alterations in motivation, self-efficacy, stress, fatigue, and social context may influence behavioral engagement and emotional reactivity over time. Ecological momentary interventions and just-in-time adaptive interventions further suggest that customized exercise prompts delivered within real-world environments may support increases in physical activity engagement, reductions in sedentary behavior, and improvements in selected cognitive outcomes, potentially through the context-sensitive reinforcement of self-regulation, neuroplasticity, and adaptive stress-response mechanisms. Collectively, these investigations provide emerging evidence supporting the potential role of exercise-oriented digital interventions as a bio-digital framework for promoting mental and cognitive well-being in aging populations via real-time monitoring and adaptive behavioral assistance.

## 1 Introduction

Population aging has emerged as one of the most pressing global public health dilemmas of the twenty-first century, characterized by a notable increase in age-related cognitive deterioration, depressive manifestations, social seclusion, and diminished physical activity among the elderly population (1). Cognitive deficits and affective disorders frequently coexist in geriatric demographics and significantly contribute to a decline in quality of life, functional impairments, heightened healthcare demands, and increased risk of mortality (2). Epidemiological research demonstrates that depressive symptoms impact a substantial segment of adults aged over 60, while the prevalence of mild cognitive impairment and dementia continues to escalate globally in tandem with increasing life expectancy (3). Simultaneously, physical inactivity and extended periods of sedentary behavior have become exceedingly common among older adults, which further intensifies neuropsychiatric susceptibility and accelerates functional deterioration (4). These interrelated issues underscore the pressing necessity for scalable, accessible, and individualized preventive interventions that can effectively bolster both mental health and cognitive fortitude in the later stages of life. Physical exercise is a well-established strategy for improving cognitive function, sleep quality, and emotional well-being in older adults. However, translating these clinical benefits into sustained behavior change in real world settings remains difficult. Traditional exercise programs frequently struggle with low long term adherence, access barriers, and a lack of personalization tailored to the fluctuating physical and emotional states of aging individuals.

Recent advancements in digital health technologies have engendered novel avenues to mitigate these constraints through the incorporation of mobile health (mHealth), wearable sensors, ecological momentary assessment (EMA), and ecological momentary interventions (EMI) into exercise-oriented mental health frameworks (5). mHealth platforms facilitate the real-time surveillance of physical activity, physiological indicators, sleep patterns, and behavioral tendencies via smartphones and wearable apparatus, thereby promoting ongoing data acquisition beyond controlled laboratory settings (6). EMA methodologies permit the recurrent real-time evaluation of mood, stress, cognition, and contextual experiences within naturalistic environments, thus diminishing recall bias and capturing temporal fluctuations in psychological states (7). Concurrently, EMIs strategies can provide tailored behavioral prompts, motivational feedback, and adaptive exercise recommendations precisely at moments when support is most essential (8). Collectively, these technologies have the potential to endorse more personalized, context-sensitive, and scalable interventions for elderly populations. Emerging research indicates that the amalgamation of exercise with digital behavioral monitoring has the potential to improve adherence, self-regulation, emotional insight, and responsiveness to interventions (9). Nevertheless, the extant literature remains disjointed across a multitude of academic disciplines, encompassing gerontology, psychiatry, behavioral medicine, digital health, neuroscience, and rehabilitation sciences. Existing reviews have typically concentrated on exercise and aging, digital mental health interventions, wearable technology, or cognitive outcomes in isolation, frequently neglecting to rigorously analyze how exercise-oriented digital interventions that incorporate EMA and EMI frameworks may collaboratively affect mood regulation and cognitive health among older adults. Moreover, numerous prior reviews predominantly prioritize technological feasibility or the promotion of physical activity in isolation, while offering insufficient discourse on mechanistic pathways, ecological validity, personalization, behavioral sustainability, and the translational implications for clinical practice (10).

The current narrative review is thus essential to synthesize these swiftly advancing yet fragmented domains of research into a cohesive conceptual framework. In contrast to previous reviews, this article distinctly centers on the convergence of physical exercise, digital health ecosystems, EMA methodologies, and cognitive-mental outcomes among the elderly population. Conversely, exercise-oriented digital interventions have the capacity to concurrently address issues of physical inactivity, social isolation, sleep disorders, cognitive deterioration, and emotional dysregulation within ecologically relevant frameworks. Empirical evidence derived from community-based and remote interventions further implies that the integration of exercise promotion with real-time digital engagement might enhance motivation, behavioral consistency, and accessibility for older adults who are either unable or disinclined to engage in conventional supervised programs. In summary, exercise-focused digital health methodologies that encompass mHealth technologies, wearable instruments, EMA, and EMI strategies may signify a groundbreaking framework for enhancing mental health and cognitive functioning throughout the aging process. The amalgamation of tailored behavioral surveillance with dynamic exercise regimens presents prospects for bolstering adherence, ecological validity, and personalized care, while simultaneously addressing the intricate biopsychosocial factors associated with neuropsychiatric decline in the context of aging. This review aspires to furnish a thorough and clinically pertinent synthesis of contemporary understanding, while delineating prospective research priorities imperative for the establishment of scalable, evidence-informed, and precision-targeted interventions for the elderly population. To synthesize these fragmented domains and establish a cohesive conceptual framework, this review adopts a narrative methodology, mapping the intersection of mobile health, EMA, and exercise for older adult cognitive and mental well-being.

## 2 Methodology and search strategy

This narrative review was undertaken to synthesize contemporary evidence concerning exercise-oriented digital interventions incorporating mHealth, EMA, and EMI for improving mental and cognitive health in older adults.

A narrative review design was chosen rather than a systematic or scoping review because of the nascent and highly heterogeneous nature of this multidisciplinary field. Systematic reviews are generally best suited to focused clinical questions involving relatively standardized interventions and outcome measures. In contrast, the literature in this area spans multiple disciplines including digital engineering, gerontology, sports medicine, psychology, and psychiatry, and exhibits substantial heterogeneity in study designs, intervention modalities, digital technologies, and outcome measures. Accordingly, a narrative approach provided the methodological flexibility required to integrate this diverse body of evidence into a coherent conceptual framework, which was the primary objective of the present review. Electronic literature searches were conducted on May 30, 2026, covering all records indexed from database inception through May 30, 2026. The search was performed across PubMed/MEDLINE, Scopus, Web of Science, and Google Scholar. Search strategies were adapted to the indexing system and syntax of each database while maintaining the same conceptual combination of keywords. Controlled vocabulary (e.g., MeSH terms, where applicable) was combined with free-text keywords using Boolean operators (AND, OR). A representative search strategy was as follows:

(“older adults” OR “elderly” OR “aging” OR “aged 50+”) AND (“exercise” OR “physical activity” OR “aerobic training”) AND (“mHealth” OR “mobile health” OR “wearable” OR “smartphone” OR “ecological momentary assessment” OR “EMA” OR “ecological momentary intervention” OR “EMI” OR “just-in-time adaptive intervention” OR “JITAI”) AND (“mental health” OR “depression” OR “cognition” OR “cognitive function” OR “executive function”).

Search filters were applied to restrict results to peer-reviewed original research articles published in English and involving human participants aged 50 years and older. The search strategy incorporated terms related to older adults, exercise and physical activity, digital health technologies (including mHealth, wearable devices, and smartphones), and real-time assessment or intervention approaches such as EMA, EMI, and just-in-time adaptive interventions (JITAIs), together with relevant mental health and cognitive outcomes including depression, affect, cognition, and executive functioning.

Google Scholar was used as a supplementary search source to identify potentially relevant studies that may not have been indexed in the primary databases and to verify the completeness of the literature. Screening of Google Scholar results was limited to the first 200 records ranked by relevance, after which no additional eligible studies meeting the predefined eligibility criteria were identified. Studies were considered eligible if they met the following criteria: (1) randomized controlled trials, pilot or feasibility studies, or longitudinal observational studies using EMA or related digital monitoring approaches; (2) interventions involving structured aerobic, resistance, multicomponent, mind-body, or walking-based exercise supported by mHealth applications, wearable activity trackers, or EMI approaches; and (3) outcomes categorized as cognitive (e.g., executive function, global cognition, dual-task performance), psychological (e.g., depressive symptoms, affect, fear of falling), or behavioral (e.g., daily step counts and sedentary behavior).

Studies published in languages other than English, studies focused exclusively on pharmacological interventions, and studies lacking a physical activity component were excluded. Following the database searches, all retrieved records were imported into EndNote for reference management and duplicate removal. A structured study selection process was then undertaken, consisting of duplicate removal, title and abstract screening, and full-text assessment for eligibility. In total, 797 records were identified through database searching. After removal of 62 duplicate records, 735 unique records remained for title and abstract screening. Following title and abstract screening, 607 records were excluded. The remaining 128 articles underwent full-text eligibility assessment, of which 111 were excluded for not meeting the predefined eligibility criteria. Ultimately, 17 studies met the eligibility criteria and were included in the final narrative synthesis.

Although this review was conducted as a narrative review rather than a systematic review and therefore did not follow the PRISMA reporting framework or employ formal dual-reviewer screening, the study identification and selection process is explicitly reported to improve methodological transparency and reproducibility. Final study inclusion was guided by thematic relevance, methodological diversity, and each study’s contribution to the conceptual objectives of the review. Only original human studies meeting the predefined eligibility criteria were included in the primary narrative synthesis. Systematic reviews, meta-analyses, clinical trial protocols, and other secondary sources were cited, where appropriate, solely to provide background information, support mechanistic discussions, identify knowledge gaps, or contextualize the evidence. These sources were not considered eligible studies and were not included in the primary narrative synthesis.

## 3 Exercise, mHealth technologies, and mental-cognitive health in older adults

mHealth technologies have emerged as promising tools for supporting the implementation of exercise interventions for geriatric populations by facilitating remote surveillance, personalized feedback, and immediate behavioral assistance. Smartphone applications, wearable sensors, smartwatches, and online exercise platforms can provide opportunities to monitor physical activity levels, heart rate, sleep patterns, and sedentary behaviors while concurrently delivering motivational cues and customized exercise regimens (9). These technological advancements enable home-based interventions that may mitigate obstacles related to transportation constraints, social isolation, and diminished access to supervised rehabilitation services (11). Notably, mHealth-integrated exercise interventions may incorporate EMA and EMI, thereby allowing for exercise recommendations to be dynamically modified in response to mood variations, stress levels, fatigue, or situational environmental influences (8). Furthermore, nascent evidence indicates that the integration of digital exercise coaching with behavioral self-monitoring may enhance participant engagement and support sustained lifestyle changes among older adults (12) (**Table 1**, **Figure 1**).

**Table 1:** Effects of exercise, mHealth, telerehabilitation, and technology-assisted interventions on cognitive function, depression, physical function, and healthy aging in older adult.

Population & sample	Intervention	Technology/mHealth component	Duration & frequency	Cognitive outcomes	Depression/Psychosocial outcomes	Physical outcomes	Findings	Limitations	Relevance to aging & mHealth	Ref
Healthy older adults ≥65 years; n=27 (Intervention=16, Control=11)	Intervention: chair-based exercise + cognitive training via synchronous telerehabilitation. Control: brochure-based chair exercises	Synchronous telerehabilitation (online real-time sessions)	8 weeks; chair exercises 2 days/week + cognitive training 1 day/week	Significant improvements in Hodkinson Mental Test and Mini-Mental State Examination in intervention group	Geriatric Depression Scale-Short Form assessed, but no major superiority between groups; Successful Aging Scale improved in control group	Improvements in Berg Balance Scale and chair-stand test in intervention group; improved TUG and dual-task TUG in control group	Both telerehabilitation and brochure-based chair exercises improved cognition and lower-extremity function; no superiority between groups	Small sample size; short intervention; cognitive training only once weekly may have been insufficient	Demonstrates feasibility of remote exercise delivery for cognitive aging; suggests low-cost home-based interventions may support healthy aging and cognitive preservation	(25)
Community-dwelling older adults ≥65 years with history of falls; n=45	Dual-task training integrating balance, strength, and cognitive exercises	Mobile app–based DT training with blended supervised/self-directed model	24 weeks; Phase 1: weekly supervised + 2 home sessions/week; Phase 2: 3 self-directed home sessions/week	Cognitive training integrated into dual-task exercise; aimed to improve cognitive-motor integration and mobility	Moderate-to-high satisfaction; social connection and tailored exercises improved adherence; no direct depression outcomes measured	Improved feasibility for fall prevention, mobility, balance, and strength training; no adverse events	Program was feasible, acceptable, and scalable beyond clinical settings; strong app engagement (95%) and retention (76%)	Nonrandomized design; no control group; feasibility focus rather than efficacy	Highlights importance of mobile health and digital tools for long-term healthy aging, fall prevention, and cognitive-motor training in older adults	(16)
Sedentary older adults; mean age 69.9 ± 3.7 years; n=49 (Experimental=21, Control=28)	Multidomain walking training program including endurance, strength, and dual-task cognitive exercises	mHealth-supported walking training program in outdoor autonomous settings	6 weeks; progressive intensity and volume	Executive function preserved in intervention group while declining in control group	Psychosocial measures assessed but no significant changes observed	Significant improvements in lower limb strength, walking speed, and cardiorespiratory fitness	mHealth-supported multidomain walking improved physical fitness and protected executive function	Short intervention period; relatively small sample; psychosocial effects not significant	Supports mobile-health–assisted exercise as a scalable strategy for preserving cognition and promoting healthy aging in community-dwelling older adults	(17)
Older adults with mild cognitive impairment or mild dementia; n=60; mean age 76.1 years	Group A: smart band + walking prescription; Group B: smart band only; Group C: walking education	Smart band telemonitoring and mHealth walking prescription	24 weeks; monthly coaching sessions	MMSE scores remained stable in Groups A and B, while Group C showed cognitive decline at 12 and 24 weeks	Depressive symptoms assessed as secondary outcomes; no major significant findings reported	Significant increase in daily steps in Group A at 12 and 24 weeks; Group B improved temporarily; Group C declined	Walking prescriptions supported by mHealth effectively increased physical activity and helped preserve cognitive status	Unblinded assessment; relatively small sample; limited depression-related outcomes	Demonstrates strong potential for wearable-based interventions to support cognition and mobility in cognitively impaired older adults	(13)
Community-dwelling prefrail older adults; randomized n=36	CogXergaming tele-exercise versus Matter of Balance tele-sessions	Gaming-based telehealth cognitive-motor exercise platform	6 weeks; 3 sessions/week; 90 minutes/session	Cognitive-motor integration targeted indirectly through exergaming	Improved self-efficacy and confidence related to balance and mobility; fear of falling indirectly addressed	Significant improvements in dynamic balance, gait, and muscle strength in CogXergaming group	Gaming-based tele-exercise is feasible and may improve physical functioning in prefrail older adults	Small pilot sample; short duration; cognitive outcomes not directly quantified	Supports gamified telehealth exercise as an engaging strategy to promote healthy aging and reduce frailty risk	(21)
Low-income community-dwelling older adults; predominantly Latino; n=13; mean age 76.4 years	FITxOlder Chinese mind-body exercise (Five Animal Frolics) led by community health worker	Mobile health-facilitated home practice support	12 weeks; supervised twice weekly + home practice up to ≥3 times/week	Cognitive function assessed with small-to-medium positive effects	High enjoyment, acceptability, social engagement, and perceived health benefits; improved quality of life indicators	Significant improvements in sit-to-stand performance and physical health survey scores	Mobile health-supported culturally tailored mind-body exercise was feasible, acceptable, and promising for healthy aging	Very small sample; no control group; preliminary feasibility design	Highlights importance of culturally adapted mobile-health exercise programs for underserved aging populations	(26)
Older low-income Latino adults; n=49	FITxOlder Chinese Qigong mind-body exercise led by Community Health Workers (CHWs)	Mobile technology-facilitated exercise support	16 weeks; weekly exercise goals	Modest improvements in cognitive function	Improved quality of life and healthy aging engagement; strong participant retention and adherence	Supportive trends for physical function improvements	Program was feasible, culturally acceptable, and safe for underserved older adults	COVID-19 disrupted implementation; modest intervention effects; feasibility focus	Demonstrates culturally tailored mobile-health exercise strategies for healthy aging in minority populations	(27)
Older adults ≥60 years with fall history; n=30	Activity tracker + multicomponent home exercise vs tracker only	Wearable activity tracker with monitoring and feedback	12 weeks	Montreal Cognitive Assessment assessed; no major significant cognitive changes reported	Significant reduction in fear of falling; depression assessed with Short Geriatric Depression Scale	Improved TUG, Short Physical Performance Battery, endurance, and mobility, especially in AT+EX group	Wearable technologies improved fear of falling and physical function even without additional structured exercise	Small sample; no major between-group differences	Supports wearable monitoring as a scalable strategy for maintaining function and confidence in aging populations	(14)
Sedentary adults ≥50 years; n=96 randomized	Smartphone walking app with or without behavioral support features	StepMATE smartphone app	4 weeks; self-directed daily walking goals	Significant cognitive improvement from pre- to posttest	Increased walking associated with improved mood and energy; stronger effects in women and older adults	Increased daily step counts from baseline	Walking combined with app-based self-monitoring improved activity, cognition, mood, and energy	Short duration; limited long-term follow-up; additional app features did not enhance outcomes	Highlights psychological and cognitive benefits of app-supported walking in sedentary aging adults	(15)
Healthy community-dwelling older adults; n=48	Motor imagery involving imagined walking, imagined talking, and imagined walking while talking	Telephone-based remote intervention	3 months	Secondary outcomes included cognitive performance during talking and dual-task walking; aimed to improve functional neuroplasticity	Potential psychosocial benefit through enhanced mobility and confidence; depression not directly assessed	Primary outcomes focused on gait speed during walking and walking while talking	Study designed to evaluate feasibility and efficacy of remote motor imagery training for gait and cognitive-motor function	Pilot protocol; efficacy outcomes pending or exploratory	Suggests low-cost remote cognitive-motor interventions may support mobility and neuroplasticity during aging	(28)
Older adults; Phase A n=18; Phase B n=11	Home-based balance training with optional cognitive training modules	Telerehabilitation system (Stability + ActiveCOG modules)	Two 4-week phases	Significant MMSE improvement in combined balance+cognitive training group	Improved usability and confidence with home training; depression not directly measured	Significant balance improvements in intervention groups	Combined motor-cognitive telerehabilitation showed clinically relevant improvements and high feasibility	Small sample size; short intervention duration; between-group differences nonsignificant	Supports home-based telerehabilitation systems for cognitive and physical healthy aging	(29)
Older adults ≥60 years with cognitive frailty; n=33 randomized	Brisk walking combined with behavior change intervention	Smartphone-assisted intervention using Samsung Health and WhatsApp	Monitored walking and activity compliance	Significant cognitive improvement observed in both groups	Potential psychosocial improvements through increased activity and engagement; depression not primary outcome	Significant improvements in frailty reduction, walking time, step count, cadence, and MVPA in intervention group	mHealth intervention enhanced adherence and improved physical activity sufficient to reduce cognitive frailty	Small sample; open-label design; preliminary findings	Demonstrates strong promise of smartphone-based exercise interventions for cognitive frailty and healthy aging	(18)

mHealth, mobile health; MMSE, Mini-Mental State Examination; HMT, Hodkinson Mental Test; DT, dual-task; TUG, Timed Up and Go; BBS, Berg Balance Scale; MVPA, moderate-to-vigorous physical activity; QoL, quality of life; CHW, Community health worker; AT+EX, activity tracker plus exercise; AT, activity tracker; FES-I, Falls efficacy scale-international; ABC, Activities-specific balance confidence; MOB, Matter of balance; RCT, randomized controlled trial; MoCA, Montreal cognitive assessment; NHS, National health service; OPQOL-Brief, Older people’s quality of life-brief; FSST, Four square step test; POMA, Performance oriented mobility assessment; MCI, mild cognitive impairment; ActiveCOG, Active cognitive training module.

**Figure 1:**
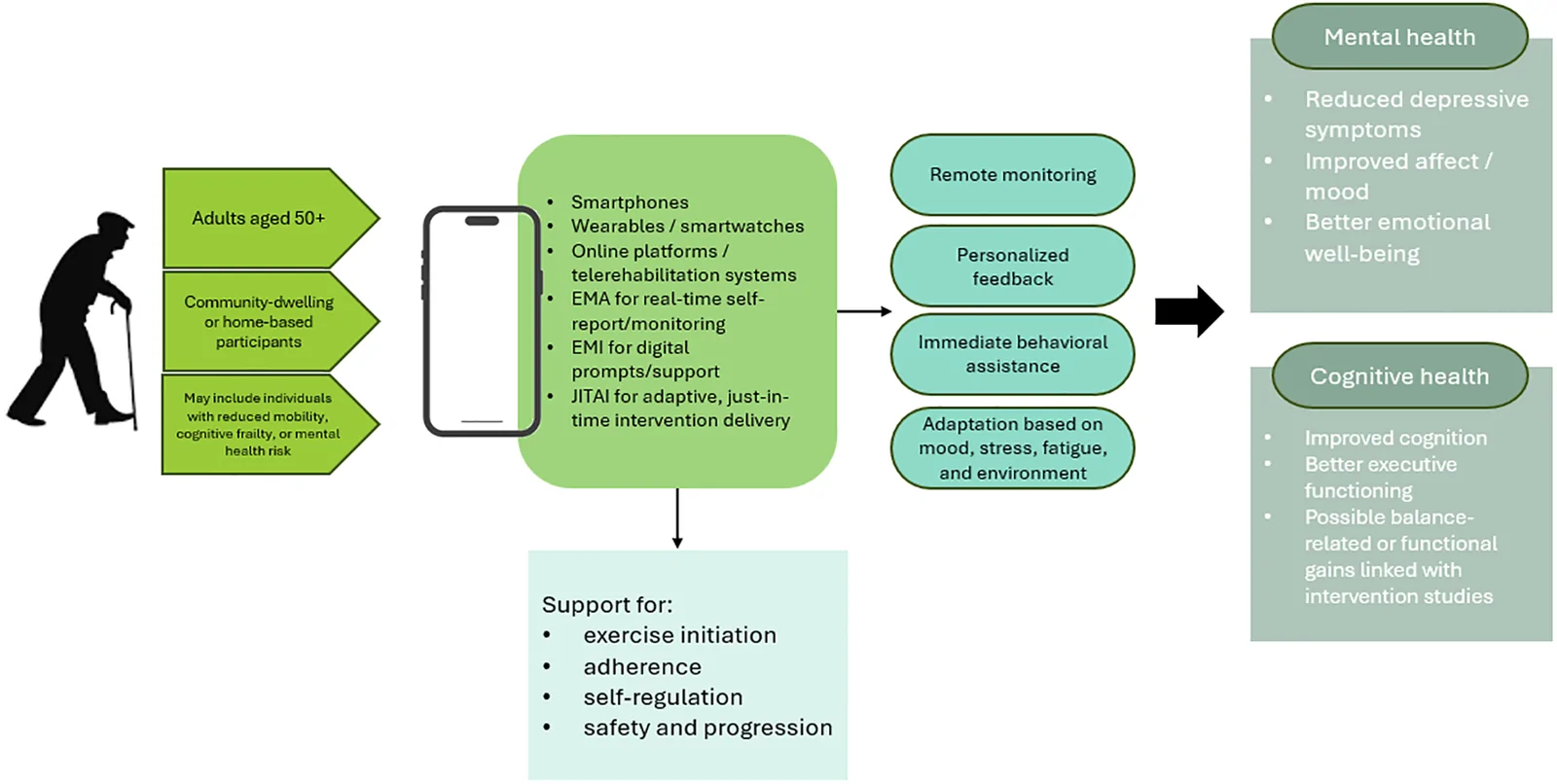
Conceptual model of exercise-based mHealth interventions for older adults.

Digital tools such as smartphones, wearables, online platforms, EMA, EMI, and JITAIs support exercise participation through remote monitoring, personalized feedback, and real-time adaptive assistance. These mechanisms may improve adherence to physical activity and contribute to better mental health outcomes, including reduced depression and improved affect, as well as cognitive outcomes such as improved cognition and executive functioning.

### 3.1 Wearable devices and activity trackers

Wearable tracking devices, smartwatches, and pedometers provide objective measures of physical activity patterns and opportunities for behavioral self-monitoring in daily life. In a 24-week randomized controlled trial comprising 60 older adults with mild cognitive impairment or mild dementia (mean MMSE 20.7 ± 4.0), the feasibility and potential benefits of mHealth-based walking prescriptions were evaluated in comparison with wearable monitoring alone and monthly walking education. Group A, which received tailored step objectives through a smart band coupled with coaching, demonstrated a statistically significant augmentation in daily steps at both the 12- and 24-week intervals (β=2205.88, P = .001; β=2194.63, P = 0.015), suggesting sustained improvements in physical activity engagement over the intervention period. Conversely, the monitoring-only cohort exhibited only a transient improvement, while the education group displayed a decline in activity over the study period, suggesting that information-based approaches alone may have limited effectiveness for maintaining behavioral change. Cognitive outcomes remained stable within the active mHealth groups; however, a decline was observed in the control group, although these alterations were not subjected to mechanistic evaluation. Considering the reliance on unblinded assessments, modest sample size, and limited cognitive endpoints, the findings should be regarded as exploratory in nature (13).

In a 12-week experimental trial involving 30 older participants with a documented history of falls (mean age 74.0 ± 6.4 years), the potential effects of combining wearable activity monitors with a comprehensive home-based exercise regimen were evaluated against a condition employing self-monitoring via the trackers alone. The intervention cohort (AT+EX) exhibited significant intra-group enhancements in fear of falling, as measured by the Activities-specific Balance Confidence (ABC) scale (P = 0.002) and the Falls Efficacy Scale-International (P = 0.01), as well as improvements in physical function, including assessments such as the Short Physical Performance Battery, Timed Up and Go, and step test performance. The group utilizing only the tracker also evidenced advancements in the Timed Up and Go test and modest decreases in fear of falling, suggesting that self-monitoring alone may contribute to some degree of behavioral modification. Nonetheless, no statistically significant differences between the groups were identified, and daily step counts remained relatively consistent across both conditions (approximately 10,000 steps per day), indicating a limited additive impact of structured exercise interventions compared with monitoring alone. Given the restricted sample size and the absence of differential outcomes, these findings remain exploratory in nature and underscore feasibility rather than establishing definitive efficacy (14).

In a four-week randomized pilot study involving 96 sedentary adults aged 50 years and older (mean age 61.6 ± 7.7 years), a behaviorally informed smartphone application (StepMATE) was evaluated against a basic pedometer-like model to assess walking behavior and associated psychological outcomes. Both cohorts exhibited a statistically significant increase in daily steps compared with baseline (from 3753 to 5248 steps/day; γ=0.24, P<0.001), suggesting that goal-setting and self-monitoring strategies alone may support short-term changes in walking behavior. Nevertheless, the lack of significant differences between groups implies that the supplemental app-based behavioral support did not demonstrate a measurable additional benefit in this study, potentially reflecting a ceiling effect or limited engagement with advanced features. Cognitive performance demonstrated a modest improvement across both cohorts (γ=0.17, P = 0.02), while EMA indicated that higher daily step counts were correlated with enhanced concurrent mood and energy levels, particularly among female and older participants. Given the short duration, reliance on self-reported affect, and limited differentiation between conditions, findings remain exploratory and primarily support feasibility and short-term behavioral responsiveness rather than sustained efficacy (15).

### 3.2 Mobile applications and EMA/EMI

Smartphone applications utilizing real-time prompting, EMA, and EMI may facilitate context-aware exercise delivery and ecological tracking of psychological states.

In a 24-week, single-arm feasibility investigation, a blended dual-task (DT) intervention amalgamating balance, strength, and cognitive training through a mobile application was assessed in 45 older adults residing in the community (≥ 65 years) possessing a documented history of falls. The program incorporated supervised group sessions during the initial 12 weeks, succeeded by home-based self-directed training, with the objective of evaluating real-world implementation rather than efficacy. Quantitatively, recruitment was acceptable (45/50 target), retention achieved 76%, and overall adherence was moderate (64%), with a marked increase in engagement during the supervised delivery phase (81%) compared to the home-based segment (50%), suggesting a potential decrease in adherence during unsupervised conditions. App utilization was notably high (95%), and no adverse events were documented. Qualitative insights underscored the significance of social interaction and personalized exercises as pivotal facilitators of participant engagement. Nevertheless, the variability in adherence and dependence on self-directed practice imply that the outcomes observed remain exploratory and inadequate to draw conclusions regarding mechanistic or clinical efficacy, thereby only substantiating preliminary feasibility for larger, controlled trials (16).

In a controlled six-week intervention study involving a cohort of 49 sedentary older adults (mean age 69.9 ± 3.7 years), a multidomain walking training program augmented by mHealth technology was evaluated against standard activity levels. The intervention incorporated endurance, strength, and dual-task cognitive components, which were administered through a progressively challenging outdoor training regimen. Following the six-week period, the experimental group demonstrated statistically significant enhancements in physical fitness parameters, including lower-limb strength, gait velocity, and cardiorespiratory fitness, suggesting short-term functional adaptations associated with the intervention. While executive function remained stable within the intervention cohort, a decline was observed in the control group, implying a potential protective association rather than definitive cognitive improvement. No substantial alterations were identified in anthropometric or psychosocial measures. Considering the limited duration, relatively small sample size, and lack of long-term follow-up, the results should be regarded as exploratory and primarily indicate feasibility and preliminary efficacy signals, rather than conclusive mechanistic insights regarding mHealth-facilitated multidomain exercise in the context of cognitive aging (17).

In a designed open-label randomized controlled trial involving 33 elderly individuals exhibiting cognitive frailty (median age of 71 years), an mHealth-enhanced brisk walking intervention was juxtaposed with conventional behavior change support alone to assess feasibility as well as hypothesis-generating evidence concerning cognitive and physical outcomes. The intervention amalgamated smartphone-based behavioral support (utilizing Samsung Health and WhatsApp) with standard guidance, with the objective of augmenting moderate-to-vigorous physical activity (MVPA) while simultaneously mitigating the progression of frailty. Feasibility indicators reflected moderate recruitment rates (33%), high retention (91%), and favorable adherence to monitoring and session attendance, implying robust acceptability despite the limited final sample size. Both cohorts exhibited significant enhancements in cognitive function (intervention P = 0.003; control P = 0.009), whereas only the mHealth cohort manifested significant advancements in frailty reduction, walking duration, step count, brisk walking duration, cadence, and MVPA (P ≤ 0.02-0.005). Nonetheless, the absence of assessor blinding, constrained sample size, and dependence on within-group analyses impose limitations on causal interpretation (18).

### 3.3 Artificial intelligence and smart coaching

Emerging literature highlights the expanding role of artificial intelligence (AI) and algorithmic decision-making in delivering personalized, adaptive exercise regimens for aging populations (19). AI-assisted interventions, including intelligent coaching algorithms and JITAIs, may enable dynamic adjustment of exercise recommendations based on real-time user performance, behavioral data, and available biofeedback information. These smart systems may provide advantages over conventional static digital approaches by facilitating context-aware feedback and potentially supporting motor learning, gait-related outcomes, and cognitive engagement, although comparative clinical evidence remains limited (20).

### 3.4 Gamification and exergaming

Gamification strategies integrate structured game mechanics, such as points, interactive challenges, and feedback loops, into physical movement to potentially enhance participant engagement and cognitive-motor dual-tasking.

In a research study concerning prefrail older adults residing in community settings, a 6-week gaming-based cognitive–motor tele-exercise program (CogXergaming, n=13) was evaluated against a structured control condition consisting of “Matter of Balance” tele-sessions (n=14). The intervention comprised frequent supervised sessions (3×/week) that integrated cognitive and motor training within an interactive framework, while the control condition emphasized lower-frequency education focused on balance. Feasibility outcomes demonstrated a satisfactory level of adherence (83% completion rate in the intervention group) despite the occurrence of early attrition in both experimental groups. Notable group × time interactions were identified for dynamic balance (Four Square Step Test, P = 0.03), gait performance (Tinetti scale, P = 0.05), and lower-limb strength (30-second chair stand, P = 0.03), with results favoring the intervention; however, no significant changes were observed in other measured outcomes. Considering the limited sample size, premature dropout, and brief duration of the intervention, the detected improvements should be regarded with caution as preliminary indicators of potential benefit rather than conclusive mechanistic evidence supporting exergaming-based cognitive–motor training for frailty prevention (21).

Beyond structured tele-exergaming, the broader integration of gamification elements in mobile exercise platforms may support exercise engagement, cognitive-motor involvement, and psychological well-being by transforming routine physical activity into cognitively stimulating interactive experiences (22).

### 3.5 Virtual reality

Virtual reality (VR) technologies provide immersive, sensory-rich environments that facilitate dual-task cognitive-motor exercise paradigms for older adults (23). By immersing users in controlled virtual environments requiring concurrent physical navigation and cognitive problem-solving, VR-based exergaming interventions may support cognitive-motor engagement, executive function, and psychological outcomes, although evidence for direct neuroplastic changes in older adults remains limited (24).

### 3.6 Telehealth and remote monitoring

Telehealth platforms, synchronous videoconferencing, and community health worker (CHW)-led digital interventions enable structured clinical supervision and social connectedness in home-based settings.

In a pilot randomized investigation involving 27 healthy adults aged 65 years and older, chair-based exercise integrated with synchronous telerehabilitation and cognitive training was evaluated against brochure-based exercise over an 8-week period. The intervention cohort (n=16) exhibited significant improvements in cognitive and functional assessments, including the Hodkinson Mental Test (P = 0.017), Mini-Mental State Examination (P = 0.006), Berg Balance Scale (P = 0.007), and chair-stand performance (P = 0.026). The control cohort (n=11) likewise manifested improvements in cognitive screening, mobility, and successful aging metrics. Nonetheless, no significant superiority between groups was identified (P>0.05), indicating that the exercise modality itself, rather than the method of delivery, may contribute substantially to the observed improvements. Given the limited sample size, brief intervention duration, and infrequent cognitive training (once per week), these findings should be approached with caution. The documented cognitive and functional enhancements remain preliminary and do not confirm definitive mechanistic effects of telerehabilitation-based cognitive exercise in aging populations (25).

In a 12-week single-arm feasibility investigation involving a cohort of 13 low-income, predominantly older Latino individuals (mean age 76.4 ± 7.9 years), a culturally tailored mind–body exercise regimen (FITxOlder) integrating community health worker (CHW)-led sessions with mHealth-supported home practice was assessed for feasibility and preliminary health outcomes. The intervention exhibited favorable feasibility, reflected by a 95% attendance rate, strong adherence to progressive home-based objectives, and elevated participant satisfaction, thereby indicating acceptable implementation within an underserved demographic. Quantitative analyses revealed statistically significant enhancements in lower-limb functionality (5-time sit-to-stand test, P<0.001, d=0.97) and self-reported physical health status (SF-12 physical component, P = 0.01, d=0.88), suggesting potentially meaningful short-term functional improvements. Nevertheless, other physical and cognitive assessments displayed only minor to moderate variations, and the single-arm design alongside a limited sample size restricts causal inference. Consequently, the findings remain exploratory, emphasizing feasibility rather than establishing definitive efficacy of culturally adapted mHealth-facilitated mind–body exercise interventions for promoting healthy aging (26).

In a Stage 1 feasibility investigation assessing the CHW-led, mobile technology–facilitated FITxOlder Qigong program involving 49 economically disadvantaged, community-residing Latino older adults over a duration of 16 weeks, the intervention exhibited robust implementation feasibility notwithstanding the disruptions caused by the COVID-19 pandemic. Elevated retention rates (89.3%) and moderate-to-high adherence levels (79.4% meeting or exceeding 70% of weekly exercise objectives) signified commendable acceptability and engagement among a demographic possessing limited access to structured healthy aging initiatives. Notably, no adverse events were documented, thereby reinforcing the safety of community-based delivery in real-world settings. Preliminary outcomes indicated only modest enhancements in physical and cognitive metrics, as well as quality of life, which align with the exploratory nature of this initial-phase trial and the lack of a fully powered control comparison group. Collectively, the findings remain hypothesis-generating, suggesting that culturally tailored, CHW-supported mHealth mind–body interventions are feasible; however, they necessitate larger RCTs to ascertain clinical efficacy and elucidate their role in fostering physical, cognitive, and functional aging outcomes (27).

In a single-blind pilot randomized controlled trial comprising 48 cognitively intact older adults, a 3-month telephone-mediated motor imagery intervention was designed to investigate the potential effects of cognitively simulating motor actions (including imagined ambulation, verbal communication, and dual-tasking involving walking while speaking) on gait performance and associated cognitive-motor outcomes. The study addresses clinically relevant gait dysfunction, which has been associated with adverse health outcomes, including increased morbidity, mortality, and dementia risk within aging populations; however, the exploratory nature of the intervention is underscored by the limited evidence available in healthy older adults. The primary outcomes evaluated encompassed gait velocity in both single-task and dual-task scenarios, whereas secondary outcomes focused on cognitive functioning during simultaneous motor activities and indicators of functional neuroplasticity. Given that this study was oriented toward feasibility and represented an initial phase of investigation lacking reported outcome data, the conclusions remain purely hypothesis-generating. Collectively, the protocol represents a preliminary mechanistic framework for cognitive-motor training, with the objective of exploring whether motor imagery may facilitate transferable enhancements in gait and executive functioning within aging individuals; nevertheless, the effectiveness of this approach requires evaluation in future outcome-based studies (28).

In a multicenter randomized pilot investigation conducted over two abbreviated 4-week phases, a home-based telerehabilitation system was assessed for its feasibility, safety, and preliminary impacts on balance and cognitive functions in the elderly population (Phase A: n=18; Phase B: n=11). Phase A involved a comparative analysis of a balance training module against conventional kinesiotherapy, whereas Phase B evaluated the efficacy of an integrated balance and cognitive training program (Stability + ActiveCOG) in relation to standard therapeutic interventions. Both phases exhibited significant within-group enhancements in balance (Berg Balance Scale; Phase A: P = 0.004 and P = 0.041; Phase B: P = 0.009), while Phase B further indicated an advancement in cognitive function (MMSE, P = 0.034), although no statistically significant differences between groups were identified (P≥0.05). Notably, effect sizes in the dual training phase were substantial (Cohen’s d>1.0), implying potential clinical import despite the constraints of limited statistical power. Given the very small sample sizes, short intervention duration, and absence of clear between-group superiority, findings remain exploratory and primarily support feasibility and safety rather than definitive efficacy of home-based motor–cognitive telerehabilitation in aging populations (29).

### 3.7 Critical appraisal of mHealth-, EMI-, and telerehabilitation-based exercise interventions

To accurately interpret the current literature, it is necessary to stratify the findings by their underlying study design. The highest level of evidence discussed in this review comes from a small fraction of fully powered randomized controlled trials. However, the vast majority of the included literature consists of single-arm feasibility trials, pilot studies, and observational EMA designs. While these preliminary designs are highly valuable for establishing system usability, participant acceptability, and short-term behavioral signals, they do not possess the statistical power or rigorous control groups required to confirm clinical efficacy. Consequently, the overarching strength of the current evidence primarily supports the feasibility of deploying these digital tools, rather than serving as definitive proof of their therapeutic impact.

Across these examinations, the evidence is constrained by persistently limited sample sizes (n≈11–99, frequently <50) and brief intervention durations (4–24 weeks), which diminishes statistical power and long-term inferential capabilities. The majority of the study designs were pilot RCTs or single-arm feasibility investigations, characterized by a frequent absence of blinding, thereby heightening the risk of performance and detection biases. Numerous trials employed within-group comparisons or demonstrated non-significant differences between groups, signifying a tendency toward exploratory rather than confirmatory evidence. The heterogeneity present within the populations examined (including healthy individuals, frail persons, cognitively impaired subjects, and caregivers) and the diversity of interventions (such as mHealth applications, telerehabilitation, EMI, JITAIs, and exergaming) further complicates comparability. The outcome measures predominantly focused on functional aspects (such as gait, balance, and steps), with a limited number of robust mechanistic or clinically pertinent endpoints, while cognitive effects were secondary and frequently modest or absent. Adherence and feasibility were generally found to be high, indicating acceptability; however, reporting bias and short follow-up periods restrict definitive conclusions regarding sustainability. In summary, current findings are hypothesis-generating, supporting the feasibility and potential utility of digital exercise interventions while highlighting the need for larger, well-controlled studies to establish clinical efficacy and mechanistic effects in aging populations.

### 3.8 Cognitive and mental health outcomes of digital exercise interventions

Digital exercise interventions have shown potential benefits on cognitive functioning and psychological wellness among the elderly population. Research that integrates physical activity with mHealth platforms has reported improvements in some domains of in executive functioning, attentional capacity, working memory, and processing velocity, potentially mediated through augmented neuroplasticity, cerebral blood flow, and neurotrophic signaling (30). Concurrently, digitally facilitated physical activity interventions may contribute to reductions in depressive symptoms, anxiety, perceived stress, and feelings of isolation through pathways involving improved self-efficacy, behavioral activation, and social connectedness (31). Technologies enabling real-time monitoring may further provide opportunities to identify changes in emotional states, activity patterns, and behavioral indicators associated with mental health outcomes (32). Nonetheless, the magnitude and consistency of these potential benefits remain variable across studies due to differences in intervention duration, exercise modalities, digital literacy, and adherence levels. Current evidence suggests that multimodal interventions combining exercise, behavioral feedback, and personalized monitoring may offer additional benefits compared with conventional approaches, although direct comparative evidence remains limited (33).

### 3.9 Feasibility, acceptability, and adherence in older adults

The feasibility and acceptability of mHealth-supported exercise interventions among older adults have received increasing attention in recent years; however, substantial challenges persist. A considerable number of older adults report favorable experiences with wearable technologies and mobile applications, attributed to improved self-monitoring capabilities, increased flexibility, and an enhanced sense of autonomy in regulating health-related behaviors (34). The provision of remote exercise interventions may hold particular significance for older adults who face mobility restrictions, chronic health conditions, or limited access to healthcare services (35). Nonetheless, adherence to these interventions is influenced by multiple factors, including digital literacy, user interface usability, cognitive capacity, socioeconomic status, and perceived technological burden. Interventions incorporating simplified user interfaces, personalized coaching, social support, and adaptive feedback mechanisms may be associated with improved retention and participant engagement (36). Despite promising results, long-term adherence remains variable, with dropout rates potentially escalating in instances where interventions lack a personalized approach or fail to address emotional and motivational challenges. Therefore, forthcoming programs should emphasize user-centered design principles and develop accessible technological infrastructures that cater to the diverse needs of aging populations (37).

## 4 EMA and intervention in aging populations

### 4.1 EMA for real-time behavioral monitoring

EMA has emerged as an increasingly used methodological framework for the real-time capture of behavioral, emotional, and cognitive variations in older adults within naturalistic settings. In contrast to retrospective questionnaires, EMA involves repeated assessments of mood, stress, physical activity, sleep, and contextual experiences through smartphones or wearable devices, thereby reducing reliance on retrospective recall and improving ecological validity (32). Within aging populations, EMA may facilitate a more precise monitoring of daily variability in depressive symptoms, fatigue, cognitive complaints, and social engagement, which are frequently dynamic and context-dependent (38). Moreover, EMA has the potential to identify short-term behavioral triggers and temporal patterns associated with sedentary behavior or reduced exercise participation. Recent findings suggest that incorporating EMA into exercise-based interventions may facilitate greater personalization and responsiveness by enabling adjustments based on real-time emotional and physiological conditions (8, 39).

In a 12-week randomized feasibility trial involving 57 spousally bereaved adults aged ≥60 years who were at elevated risk for depression, the comparative efficacy of digital monitoring of sleep, dietary habits, and physical activity—administered with or without health coaching—was evaluated against enhanced usual care to elucidate trajectories of depressive symptoms. The high rates of recruitment (85%), retention (88%), and adherence (90–92%) indicated favorable feasibility and acceptability of continuous behavioral tracking within a vulnerable geriatric population. Although linear mixed-effects models indicated a statistically significant overall decrease in depressive symptoms over the duration of the study, no significant interaction between time and intervention was observed, suggesting limited empirical support for the additional efficacy of coaching beyond monitoring in isolation. Considering the modest sample size, the brevity of the intervention period, and the lack of mechanistic endpoints, the observed changes remain exploratory and may reflect to non-specific engagement or regression effects rather than definitive causal intervention effects. Collectively, these findings bolster the feasibility of employing digital behavioral monitoring in the context of late-life bereavement, yet they yield only hypothesis-generating evidence regarding its potential antidepressant effects (40) (**Table 2**).

**Table 2:** Effects of exercise, ecological momentary interventions, ecological momentary assessment, and digital behavioral monitoring on cognitive function, depression, physical activity, and healthy aging in older adults.

Population & sample	Intervention	EMI/EMA/Digital component	Duration & frequency	Cognitive outcomes	Depression/Psychosocial outcomes	Physical outcomes	Findings	Limitations	Relevance to aging & mHealth	Ref
Older spousally-bereaved adults ≥60 years at high risk for depression; n=57	Digital monitoring with or without motivational health coaching versus enhanced usual care	Continuous digital monitoring of sleep, meals, and physical activity with motivational coaching	12-week intervention with 9-month follow-up	Cognitive outcomes not primary focus	Depression symptoms decreased over time; intervention feasible and acceptable; high adherence and retention	Physical activity monitored continuously	Digital monitoring and coaching were feasible and acceptable among bereaved older adults	Interaction effect on depression not significant; pilot sample size	Demonstrates feasibility of digitally supported behavioral interventions to address depression risk during aging and bereavement	(40)
Community-dwelling Belgian older adults; PA study n=88, SB study n=76; median age 72 years	Real-time monitoring of physical activity and sedentary behavior	Fitbit-triggered EMA surveys via HealthReact smartphone app	7-day continuous monitoring	No direct cognitive intervention; behavioral awareness and motivation assessed	Increased awareness and motivation for physical activity in some participants; good user acceptability	Real-time capture of PA and sedentary behavior; compliance ~80%	Sensor-triggered EMA is feasible for studying real-time behavior patterns in older adults	Technical issues; notification timing; latency problems	Highlights utility of EMA for understanding aging behaviors and designing personalized physical activity interventions	(41)
Community-dwelling Spanish-speaking Latino adults; n=39; mean age 61 years	Individualized ecological momentary intervention to replace sedentary time with physical activity	Fitbit monitoring, SMS text messaging, ecological momentary assessment, coach feedback, phone meetings	6 weeks	Significant improvement in executive function (Trail Making Test Part B)	High satisfaction and motivation; intervention well accepted	Increased step counts and reduced sedentary time	Individualized EMI improved executive function and promoted physical activity replacement behaviors	Small sample; short intervention; limited cognitive measures	Supports culturally tailored EMI approaches to improve cognition and reduce sedentary aging behaviors	(42)
Community-dwelling older adults aged 56–72 years; n=46 recruited, n=31 completed	Personalized smartphone-delivered physical activity support intervention	Fitbit activity tracker + JitaBug smartphone app delivering tailored JITAI messages based on real-time activity, time, and weather	6 weeks	Cognitive outcomes not primary focus; mood and well-being monitored through ecological momentary assessment	High participant satisfaction; mood and mental well-being assessed repeatedly; positive acceptability	Personalized PA goal support and real-time activity encouragement	Smartphone-delivered JITAI was feasible and acceptable for increasing or maintaining physical activity in older adults	Moderate completion rate; technical refinements needed; lower completion of mood/well-being assessments	Demonstrates feasibility of adaptive real-time digital behavior change systems for promoting active aging	(43)
Previously underactive family caregivers of patients with dementia; n=68 randomized	Staff-supported aerobic exercise training versus waitlist control	Ecological momentary assessment (EMA) administered 6 times/day for 7 days before and during intervention	24 weeks	Cognitive outcomes not directly measured	Significant increases in positive affect and perceived control; significant reductions in negative affect and rumination	Aerobic exercise intervention promoted regular physical activity engagement	Aerobic training substantially improved daily psychological processes and mental health indicators in caregivers	Secondary analysis; caregiver-specific population; cognition not directly assessed	Highlights role of exercise combined with EMA in improving emotional well-being and resilience during aging-related caregiving stress	(44)

EMA, ecological momentary assessment; EMI, ecological momentary intervention; JITAI, just-in-time adaptive intervention; PA, physical activity; SB, sedentary behavior; QoL, quality of life; SMS, short message service; MVPA, moderate-to-vigorous physical activity; FAST, Fitness, Aging, and STress trial; Fitbit, wearable activity tracker; PA/SB, physical activity/sedentary behavior; OR, odds ratio; CI, confidence interval.

A methodological investigation assesses the viability and analytical efficacy of sensor-activated EMA for the acquisition of real-time physical activity (PA) and sedentary behavior (SB) among community-residing older adults (PA n=88; SB n=76; median age 72 years) over a span of 7 days, utilizing Fitbit-integrated smartphone prompts. Rather than focusing on health outcomes, this study offers hypothesis-generating insights regarding measurement validity and the functionality of behavioral monitoring systems. Overall compliance with EMA was notably high (~80%); however, SB exhibited lower confirmation rates (72.4%) in comparison to PA (94.2%), suggesting lower concordance between self-reported sedentary behavior and device-derived measurements. Mixed-effects models revealed an increase in engagement over time in SB (OR = 1.59, P<0.01), while the confirmation of PA diminished with the length of the study (OR = 0.81, P = 0.02) and increased response latency (OR = 0.80, P<0.01), thereby highlighting potential temporal changes in participant responding. Simulations indicated that shorter event durations were associated with enhanced data capture. Despite the usability of the system, technical complications and failures in notifications resulted in measurement bias (41).

A pilot randomized investigation (n=39; mean age 61 ± 5.8 years) assessed a 6-week EMI aimed at substituting sedentary behavior with physical activity among Spanish-speaking Latino adults, a demographic identified as at high risk for cognitive decline associated with inactivity. The intervention integrated Fitbit-based monitoring, SMS reminders, coaching, and personalized behavioral adjustments, and was evaluated against guideline-congruent educational approaches. The results are predominantly hypothesis-generating, revealing substantial feasibility (79% adherence) and acceptability (9.4/10 satisfaction; 9.8/10 motivation), alongside modest behavioral and cognitive outcomes. In comparison to control participants, the EMI cohort exhibited an increase in weekly steps (+5543; d=0.54, P = 0.05) and a reduction in sedentary time, although the latter did not achieve statistical significance (d=0.47, P = 0.24), indicating potential transient behavioral alterations without substantial activity substitution. Notably, executive function demonstrated a significant enhancement on the Trail Making Test B (d=0.74, P = 0.01), suggesting a prospective cognitive advantage, although no changes were observed in other cognitive domains. Small sample size, short duration, and reliance on self-reported feasibility metrics limit causal inference and generalizability, warranting larger confirmatory trials (42). A feasibility study (n=46; age 56–72 years; 6-week duration) assessed a smartphone-based just-in-time adaptive intervention (JitaBug) that integrates Fitbit tracking, contextual tailoring (time, behavior, weather), and behavior change methodologies to facilitate physical activity among older adults residing in community settings. The primary objective of this research is to generate hypotheses, emphasizing the feasibility of delivery over efficacy. The overall retention rate was moderate (67%); however, system performance was notably high, achieving a successful delivery rate of 94% of messages and near-complete operational functionality of wearable and environmental data streams (>99%). Adherence levels demonstrated variability across components (voice memos 50%, mood assessments 38%), reflecting differential engagement with ecological momentary features. Notably, 77% of participants indicated their satisfaction, which suggests a favorable level of acceptability, although qualitative feedback underscored usability challenges and the necessity for interface enhancements. No significant adverse events were reported, providing preliminary information regarding safety within this feasibility context. Nonetheless, the limitations posed by missing data, incomplete engagement, and the brief duration of the intervention limit conclusions regarding sustained behavioral effects (43). A secondary analysis of the FAST randomized controlled trial (n=68; analyzed n=56) investigated the extent to which a 24-week structured aerobic exercise regimen influences ecological momentary psychological processes among physically inactive family caregivers of individuals with dementia. Through the implementation of intensive EMA sampling (comprising six evaluations per day over a span of seven days at baseline and week 24), the research yields hypothesis-generating evidence concerning affective and stress-related mechanisms rather than immediate physiological responses. In comparison to waitlist controls, the intervention cohort exhibited more pronounced enhancements in positive affect and perceived control, accompanied by decreases in negative affect and rumination, suggesting possible longer-term changes in affective processes rather than only transient mood fluctuations. Nonetheless, the absence of mechanistic biomarkers precludes a definitive causal inference regarding the underlying neurobiological pathways. Furthermore, attrition occurring between randomization and EMA completion (n=56 analyzed) introduces a potential selection bias, and the dependence on self-reported momentary states may exacerbate measurement variability (44).

### 4.2 Wearable and EMA interventions in older adults: evidence and critical appraisal

Across these pilot and randomized investigations (n≈39–68; duration 6–24 weeks), digital and exercise-based interventions exhibit a generally favorable feasibility profile; however, their causal inferences are constrained by methodological limitations. The EMI trial (n=39, 6 weeks) reported a moderate effect size for executive function (d=0.74, p=0.01), but the small sample size and abbreviated follow-up period hinder the robustness of durability conclusions. The JitaBug JITAI study (n=46, 6 weeks) demonstrated high system delivery performance (>80% successful intervention delivery); nevertheless, the incomplete engagement with EMA components (38–50%) introduces potential self-selection and measurement biases. The 24-week FAST trial (n=68, analyzed n=56) suggested possible changes in affect regulation; however, participant attrition and dependence on self-reported EMA data may diminish the internal validity of the findings. Across these studies, the unblinded design, heterogeneous nature of interventions, and absence of active control groups may increase the risk of performance bias. mHealth walking and monitoring trials (6–12 weeks, n≈30–60) have reported variable cognitive and physical outcomes, frequently exhibiting insufficient power to detect between-group differences. Overall, evidence remains exploratory, supporting feasibility and behavioral signals rather than established mechanistic or clinical efficacy, warranting larger, longer RCTs.

### 4.3 Contextual and time-varying determinants of physical activity

The physical activity behavior exhibited by older adults is influenced by dynamic contextual and temporally varying factors such as mood, meteorological conditions, pain levels, fatigue, quality of sleep, social interactions, and accessibility of the environment (45). Conventional assessment methodologies frequently fail to adequately capture these moment-to-moment fluctuations, thus constraining the comprehension of the reasons behind the considerable variability in exercise adherence among individuals and across different daily circumstances. EMA-based methodologies offer valuable opportunities to elucidate behavioral determinants in real time and to investigate how psychological and environmental contexts influence physical activity patterns (46). For instance, transient manifestations of depressive symptoms, feelings of loneliness, or suboptimal sleep may be associated with reduced exercise engagement during particular intervals, while social support and positive affect may serve to bolster motivation and adherence (47). Acknowledging these temporal and contextual influences is crucial for the formulation of adaptive interventions customized to the distinctive behavioral rhythms and functional capacities of older adults. Such methodologies may ultimately support sustained exercise participation and potentially contribute to improved mental and cognitive outcomes, although long-term effectiveness requires further investigation (48).

### 4.4 EMI and JITAI

EMIs and JITAIs signify innovative methodologies aimed at providing tailored behavioral support within real-world contexts precisely at moments when individuals exhibit heightened receptivity or vulnerability (8). Among older adults, these methodologies may facilitate adherence to exercise regimens and enhance psychological well-being by amalgamating real-time behavioral data with adaptive digital feedback mechanisms. JITAIs can incorporate data derived from wearable technology, EMA responses, and contextual sensors to deliver personalized exercise prompts, motivational communications, stress-management techniques, or reminders during episodes of inactivity or emotional turmoil (49). Such interventions could be particularly advantageous for aging demographics due to their capacity to adapt to daily fluctuations in physical abilities, mood states, and environmental factors. Notably, EMIs extend beyond fixed intervention approaches by allowing adjustment of intervention timing and content according to changing user conditions (50). While initial findings exhibit promise, additional investigation is necessary to ascertain the long-term efficacy, usability, and scalability of JITAI-centered exercise interventions across varied older populations (51).

### 4.5 Digital phenotyping: integration of wearables and EMA

Digital phenotyping is defined as the ongoing quantification of behavioral, physiological, and psychological patterns through the utilization of data derived from smartphones, wearable technologies, and various digital interactions (52). Within aging populations, the integration of wearable sensors with experience sampling methodologies presents a potentially valuable framework for examining the complex relationships among physical activity, mood, sleep, cognitive function, and daily living activities. Wearable technologies can continuously monitor heart rate, mobility, gait, sleep patterns, and sedentary behavior, while experience sampling concurrently captures subjective experiences encompassing stress, emotional states, fatigue, and cognitive complaints (53). The synthesis of these multimodal data streams may improve the characterization of behavioral and psychological changes associated with cognitive and emotional health, although its ability to predict clinically meaningful deterioration remains to be established (54). Moreover, digital phenotyping may facilitate more individualized exercise strategies that are tailored to behavioral profiles and temporal dynamics. However, ethical dilemmas pertaining to privacy, data security, technological accessibility, and algorithmic bias remain critical challenges requiring careful consideration in future research and clinical applications (55).

## 5 Mechanistic pathways linking exercise, digital monitoring, and mental health

Exercise-induced enhancements in mental and cognitive health may involve a multitude of interrelated neurobiological and behavioral mechanisms that may be further explored through the application of digital monitoring technologies. A particularly well-studied pathway encompasses neuroplasticity and the modulation of BDNF, neuronal viability, and hippocampal neurogenesis (56). The process of aging is frequently correlated with diminished neuroplastic capacity and compromised hippocampal functionality, both of which contribute to cognitive deterioration and increased susceptibility to depressive disorders. Consistent engagement in physical activity has been associated with alterations in circulating BDNF levels and adaptive structural and functional changes within brain regions pertinent to memory, executive functioning, and emotional regulation (57). Recent findings indicate that wearable technologies and real-time behavioral monitoring may assist in optimizing exercise intensity, frequency, and adherence, potentially supporting conditions that are favorable for neuroplastic adaptation in older adults. Inflammation and oxidative stress are pivotal factors in neuropsychiatric disorders associated with aging. Chronic low-grade inflammation, commonly termed “inflammaging,” correlates with elevated concentrations of pro-inflammatory cytokines, including interleukin-6 and tumor necrosis factor-α, which may hinder synaptic communication, expedite neuronal degeneration, and exacerbate depressive manifestations (58). Concurrently, excessive oxidative stress has the potential to compromise mitochondrial functionality and disrupt neuronal integrity, thereby adversely influencing cognitive processes and emotional resilience. Physical exercise induces anti-inflammatory and antioxidant responses through the modulation of immune signaling pathways, augmentation of intrinsic antioxidant defenses, and enhancement of mitochondrial efficacy (59). Digital monitoring systems may further support these processes by identifying patterns of prolonged sedentary behavior, sleep disruption, or stress-related physiological changes that may be associated with inflammatory dysregulation. Consequently, the integration of exercise interventions with behavioral and physiological monitoring may provide opportunities to better understand maladaptive patterns and personalize intervention strategies, although direct neuroprotective effects of digital monitoring remain to be established.

Another salient mechanism linking physical exercise and mental health pertains to the regulation of affective states and neural reward circuitry. Engagement in physical activity may influence dopaminergic, serotonergic, and endocannabinoid pathways that are associated with positive affect, motivation, stress resilience, and emotional processing (60). In the geriatric population, these neurochemical responses may serve to mitigate anhedonia, apathy, and depressive symptoms that are commonly observed in conjunction with aging and social isolation. Additionally, participation in exercise programs may foster enhanced self-efficacy, autonomy, and perceived behavioral control, thereby bolstering adaptive psychological conditions. Digital platforms for exercise that incorporate elements of motivational feedback, gamification, and personalized reinforcement mechanisms may enhance reward-related behavioral responses and facilitate sustained engagement in physical activity interventions (61). However, an excessive dependence on technological solutions devoid of sufficient personalization or emotional significance may lead to a diminishment of intrinsic motivation in certain individuals, thereby underscoring the necessity of integrating digital assistance with user-centered behavioral methodologies. The interplay among physical activity, affective states, and cognitive processes is increasingly acknowledged as a dynamic bidirectional feedback mechanism, rather than a simplistic linear causal framework. Elevated emotional states may augment the propensity to participate in physical exercise, whereas consistent engagement in exercise may subsequently enhance mood, attention, quality of sleep, and cognitive efficacy (62). Similarly, improvements in cognitive function may facilitate planning, self-regulation, and adherence to health-promoting behaviors, potentially creating reinforcing behavioral cycles. Digital monitoring technologies provide opportunities to examine these interactions longitudinally through continuous assessment of movement patterns, emotional states, sleep characteristics, and cognitive-related behaviors in everyday settings. Such data may support the development of adaptive interventions aimed at addressing unfavorable behavioral patterns before they become clinically significant, although their predictive and preventive capabilities require further validation (63). Empirical observations further indicate that the integration of exercise interventions with personalized digital feedback may heighten awareness of behavioral trends and reinforce long-term self-management abilities among older adults.

In summary, mechanistic evidence suggests that exercise-oriented digital interventions may influence on mental and cognitive health via interconnected neurobiological, psychological, and behavioral pathways. The convergence of neuroplastic adaptations, anti-inflammatory processes, affective modulation, and real-time behavioral surveillance provides hypothesis-generating evidence for precision health strategies in healthy aging. Nonetheless, significant variability in intervention protocols, biomarker evaluations, and digital approaches persists as a substantial limitation within the extant literature. Consequently, forthcoming investigations should emphasize longitudinal multimodal methodologies that are capable of incorporating biological, behavioral, and ecological data to enhance the comprehension of individualized responses to exercise-focused digital health interventions among aging populations.

## 6 Challenges and limitations

Notwithstanding the increasing potential of exercise-based digital interventions and EMA methodologies in older populations, numerous significant challenges and constraints persist. A predominant concern within the current literature is the prevalence of limited sample sizes and preliminary or feasibility-focused study designs. Although these investigations are instrumental in determining initial feasibility and acceptability, they consequently constrain statistical power and may limit the generalizability of findings to broader aging populations. Furthermore, considerable variability exists in EMA protocols, encompassing discrepancies in sampling frequency, duration of monitoring, types of sensors utilized, and assessment instruments employed. This inconsistency complicates direct comparisons across studies and limits the development of standardized methodological frameworks for future research.

Additionally, a critical limitation pertains to technology adherence and digital literacy among older adults. Although many studies report favorable acceptability, sustained engagement with smartphones, wearable devices, and application-based systems remains challenging, particularly among individuals with cognitive impairment, limited technological experience, or restricted access to digital resources. Furthermore, measurement bias and reactivity effects constitute important concerns within this domain. Repeated self-monitoring through EMA may inadvertently influence participants’ behaviors, potentially increasing awareness of physical activity patterns or altering mood reporting, which may affect the interpretation of natural behavioral responses. Moreover, discrepancies between self-reported EMA data and objective measurements derived from wearable technologies highlight ongoing challenges related to data validity and multimodal data integration.

Finally, a notable limitation of the existing literature is the lack of long-term follow-up data. Most interventions are relatively short-term and do not adequately determine whether behavioral or psychological changes are maintained over extended periods. In the absence of longitudinal evidence, it remains uncertain whether these digital approaches can produce sustained improvements in physical activity, cognitive functioning, and mental health outcomes among older adults.

Another major limitation of the current evidence base is the substantial heterogeneity across included studies. Target populations vary widely, encompassing healthy community-dwelling older adults, frail individuals, caregivers, and those with pre-existing cognitive impairment. Furthermore, interventions differ considerably in duration, exercise intensity, and digital technologies employed, ranging from basic smartphone step counters to complex JITAIs. Because clinical outcomes and assessment endpoints are similarly diverse, this heterogeneity limits the ability to draw generalized conclusions regarding the efficacy of digital platforms across different aging populations.

Consequently, these methodological limitations necessitate cautious interpretation of current findings. The combination of small sample sizes, short intervention periods, variable digital literacy, and attrition challenges indicates that observed behavioral or cognitive improvements should not yet be translated into definitive clinical recommendations. At present, the evidence provides support for the feasibility, participant acceptability, and preliminary potential of exercise-based digital interventions. However, current evidence does not yet confirm their clinical efficacy. Until adequately powered, long-term randomized controlled trials are completed, these digital platforms should currently be considered promising exploratory tools rather than established therapeutic interventions.

Finally, it is necessary to acknowledge the methodological limitations of this narrative review itself. Because we employed a narrative design rather than a systematic approach, the literature search and subsequent synthesis are inherently more susceptible to selection bias (64). Furthermore, our inclusion criteria restricted the search to English-language publications, which introduces a potential language bias and may have excluded relevant international findings. We also did not conduct a formal risk-of-bias or methodological quality assessment for the included studies. Future research should aim to build upon this conceptual framework through rigorous systematic reviews and meta-analyses incorporating standardized quality assessment tools to more comprehensively evaluate the clinical effectiveness and evidence quality of these interventions.

### 6.1 Data governance, privacy, and ethical considerations

Deploying digital health tools and EMA in aging populations introduces important ethical and regulatory challenges. Continuous passive sensing and location tracking may involve the collection of sensitive real-time behavioral data, which raises substantial privacy concerns regarding unauthorized third-party access, commercial data use, and limitations in user consent frameworks (55). Older adults, particularly those experiencing mild cognitive impairment or early-stage dementia, may face difficulties fully understanding dynamic data-sharing permissions or consenting to continuous surveillance (65). Furthermore, data storage architectures should adhere to relevant regulatory standards, such as the General Data Protection Regulation in Europe or the Health Insurance Portability and Accountability Act in the United States, to support secure data transmission and appropriate protection of personal information. Safeguarding participant autonomy while maintaining real-time digital monitoring would benefit from transparent data governance policies, clear opt-out mechanisms, and user-centered design approaches that prioritize participant dignity.

### 6.2 Digital inequity, algorithmic bias, and implementation barriers

A critical barrier to the widespread adoption of exercise-based digital health interventions is the risk of exacerbating digital inequity. Access to smart devices, reliable high-speed internet, and digital literacy varies widely across socioeconomic, racial, and geographic demographics (66, 67). Interventions that rely heavily on advanced smartphone interfaces or expensive wearable devices may unintentionally exclude vulnerable, low-income, or rural older adult populations, potentially widening existing healthcare disparities. Additionally, machine learning algorithms and JITAIs trained on limited or homogeneous datasets may introduce algorithmic bias (68). If predictive models for physical activity patterns or affective states are primarily developed using younger or healthier populations, their performance may be reduced when applied to older adults with mobility limitations, chronic diseases, or diverse functional profiles. Addressing these implementation challenges requires the development of accessible, low-bandwidth, culturally adapted digital tools and validation of predictive algorithms across diverse, real-world aging populations.

## 7 Future directions

Future inquiries ought to focus on developing more sophisticated, scalable, and clinically relevant frameworks to further improve the personalization and applicability of exercise-based digital interventions in older adults. A pivotal avenue of exploration is the amalgamation of artificial intelligence (AI) with JITAI to facilitate highly individualized, context-sensitive support systems. Through the utilization of real-time data derived from wearable sensors and EMA, AI-enhanced models may help identify individualized behavioral patterns, fluctuations in mood, and contextual factors, potentially supporting the delivery of interventions at more appropriate moments to facilitate behavioral change. Another critical priority is the standardization of EMA methodologies. Presently, existing studies exhibit considerable variability in terms of sampling frequencies, measurement instruments, and analytical methodologies, which may limit reproducibility and complicate comparisons across studies. The establishment of consensus guidelines for EMA design, reporting, and data integration could improve methodological consistency and facilitate future advancements in this field. Moreover, there exists a distinct necessity for larger-scale randomized controlled trials with longer follow-up periods to further evaluate the effectiveness, sustainability, and clinical relevance of these interventions. The majority of current evidence derives from limited pilot studies, and rigorous clinical trials are needed to better determine the potential causal effects of these approaches on mental health, cognitive functioning, and physical activity outcomes. Subsequent research endeavors should also emphasize the integration of these digital methodologies within clinical psychiatry and geriatric care environments. The incorporation of mHealth and EMA-based instruments into standard healthcare practices may provide opportunities for continuous monitoring and more personalized treatment adjustments, although their feasibility and clinical utility require further investigation. Ultimately, the advancement of scalable, economically viable digital therapeutics specifically designed for aging populations may represent an important step toward translating emerging evidence into meaningful public health applications.

## 8 Conclusion

In conclusion, exercise-based digital interventions incorporating mHealth and EMA show promising feasibility and potential to support improvements in daily physical activity and functional outcomes among older adults. However, current evidence regarding their direct clinical efficacy on cognitive and mental health outcomes remains preliminary and heterogeneous. Until adequately powered, long-term randomized controlled trials with appropriate active control groups, rigorous methodological quality assessments, and robust clinical outcomes are conducted, these digital methodologies should be viewed as promising exploratory and supportive tools rather than fully established clinical therapies.
