# Supplementary material for: From movement to mood regulation: exercise-based digital interventions using mobile health and ecological momentary assessment for mental health and cognitive function in older adults: a narrative review
Source: Front Psychiatry. 2026 Sep 30;17:1895581. doi: 10.3389/fpsyt.2026.1895581 (PMC13669503; doi:10.3389/fpsyt.2026.1895581)
Supplement: Supplementary file 1 [file Table1.docx]

**Appendix s1: Comprehensive database search strategies**

## This appendix provides the complete, database-specific search strings and syntax used to identify literature for this narrative review.

## Search timing and coverage period. The electronic searches were executed on May 30, 2026. For each database, the publication coverage period was from database inception to the search date (May 30, 2026). Search results were limited to English-language records, and human/age filters were applied where available

## 1. PubMed / MEDLINE

**Search String:**

Plaintext

(("Older Adults"[Mesh] OR "Aged"[Mesh] OR "Aging"[Mesh] OR "elderly" OR "older adult*" OR "aged 50+" OR "middle-aged and older") AND ("Exercise"[Mesh] OR "Physical Fitness"[Mesh] OR "Walking"[Mesh] OR "exercise" OR "physical activity" OR "aerobic training" OR "resistance training") AND ("Mobile Applications"[Mesh] OR "Cell Phone"[Mesh] OR "Telemedicine"[Mesh] OR "mHealth" OR "mobile health" OR "wearable electronic devices" OR "smartphone" OR "ecological momentary assessment" OR "EMA" OR "ecological momentary intervention" OR "EMI" OR "just-in-time adaptive intervention" OR "JITAI") AND ("Mental Health"[Mesh] OR "Cognition"[Mesh] OR "Depression"[Mesh] OR "Executive Function"[Mesh] OR "mental health" OR "depression" OR "cognition" OR "cognitive function" OR "executive function" OR "mood regulation"))

## **Filters applied: Humans; English language; Age: 50+ years (where available/applicable).**

## 2. Scopus

**Search String:**

Plaintext

TITLE-ABS-KEY(("older adult*" OR "elderly" OR "aging" OR "aged 50+") AND ("exercise" OR "physical activity" OR "aerobic training" OR "resistance training") AND ("mHealth" OR "mobile health" OR "wearable*" OR "smartphone*" OR "ecological momentary assessment" OR "EMA" OR "ecological momentary intervention" OR "EMI" OR "just-in-time adaptive intervention" OR "JITAI") AND ("mental health" OR "depression" OR "cognition" OR "cognitive function" OR "executive function")) AND (LIMIT-TO (PUBSTAGE, "final")) AND (LIMIT-TO (LANGUAGE, "English"))

## **Filters applied: Document type: Article or Review; Language: English.**

## 3. Web of Science (Core Collection)

**Search String:**

Plaintext

TS=(("older adult*" OR "elderly" OR "aging") AND ("exercise" OR "physical activity" OR "aerobic training") AND ("mHealth" OR "mobile health" OR "wearable*" OR "smartphone" OR "ecological momentary assessment" OR "EMA" OR "ecological momentary intervention" OR "EMI" OR "just-in-time adaptive intervention" OR "JITAI") AND ("mental health" OR "depression" OR "cognition" OR "cognitive function" OR "executive function"))

## **Filters applied: Language: English; Document types: Article or Review.**

## 4. Google Scholar

Role of Google Scholar. Google Scholar was used as a supplementary source to (i) identify potentially relevant items not indexed in the databases above and (ii) support citation chasing (backward/forward screening) of key included papers.

Search approach. Because Google Scholar has limited reproducible filtering and exporting functionality, we used a structured keyword approach and screened results ranked by relevance.

Search strings (run separately):

- All in title: exercise OR "physical activity" AND "mobile health" OR EMA OR "ecological momentary assessment" AND "older adults" OR aging
- "ecological momentary intervention" AND exercise AND (cognition OR "mental health") AND ("older adults" OR aging)
- "just-in-time adaptive intervention" AND "physical activity" AND ("older adults" OR aging)

Screening and stopping rule. For each query, we screened the first 200 results sorted by relevance, after removing obvious duplicates across queries. Screening stopped at 200 per query because relevance declines substantially beyond this range and because Google Scholar ranking is not stable enough to support exhaustive reproducible retrieval; thus, it was used primarily for supplementary identification rather than comprehensive capture.
